# Sympathy between the Auditory Canal and the Larynx

**Published:** 1866-10

**Authors:** 


The September number of the *Cincinnati Journal of Medicine* contains a paper by Dr. J. F. Hibberd, of Richmond, Ind., from which we reprint the following sentences:

Just now an active effort is being made to introduce into this city another nostrum called "Fluid Extract of Sarsaparilla with Iodide of Lime." This last article may be an excellent remedial agent, but it is presented surrounded by the declaration that large numbers of the most eminent physicians have testified to its worth; by the promise that it is admirably adapted to the use of children in chronic diseases, and by all those special pleadings that nostrum makers know so well how to apply to cajole the public into buying their wares.

This Iodide of Lime is one of the products of the laboratory of J. R. Nichols & Co., who have a very specioas and Oily

Gammon way of presenting their preparations to the profession. For some years this house has been making and vending an "Elixir of Bark and Iron," the great merit of which they claimed, was, that it contained the protoxide of iron, whereas it contains no such ingredient. Of this fact I have long been satisfied, but to fix the affair with chemical certainty, Dr. Weist, at my request, during the present week, examined a specimen that I presented him, and neither by the test paraded by the proprietors, nor by other tests, could any protoxide of iron be detected.*

Within the last forty-eight hours, while I was preparing this paper, the traveling agent of this same house laid upon my table a circular, one side of which is devoted to puffing the iodide of lime, and the other side is taken up with an essay on "Opium and its Alkaloids," leading to the announcement that the Tinctura Opii Deodorata of the Pharmacopœia is an excellent preparation, but that its title is a misnomer, and that they prepare the article in a superior manner, and *propose to vend it under the name of "Infusum Opii Deodorata."* Such brazen impudence is past being tolerable, and ought to rule the house of its perpetrators from the catalogue of reputable pharmaceutists,—and prevent any of their preparations from being found in any respectable drug store.

* Dr. Hibberd has written a letter, received since his paper was presented, wherein he states that Dr. Weist has carefully examined a number of specimens of the preparation, and finds them to contain a proto-salt and a sesqui-salt of iron in varying quantities, the quantity of the one or the other salt probably depending upon the age and exposure of the particular specimen examined.

